# Relationship Between the 2019 Ridgecrest, California, *M*_W_7.1 Earthquake and Its *M*_W_6.4 Foreshock Sequence

**DOI:** 10.3390/e27010016

**Published:** 2024-12-28

**Authors:** Jianchang Zheng, Zhengshuai Zhang, Xiaohan Li

**Affiliations:** 1 Department of Mathematics and Statistics, University of Otago, Dunedin 9054, New Zealand; 2 Shandong Earthquake Agency, China Earthquake Administration, Jinan 250102, China; zzs@cumt.edu.cn (Z.Z.); byfeiyu@mail.ustc.edu.cn (X.L.)

**Keywords:** earthquake clusters, foreshock, aftershocks, earthquake sequence, statistical seismology

## Abstract

The 2019 Ridgecrest *M*_W_7.1 earthquake has received significant attention due to its complex fault activity. It is also noticeable for its *M*_W_6.4 foreshock sequence. There are intricate dynamic relationships between earthquakes in such vigorous sequences. Based on the relocated catalogue, we adopt the nearest neighbour algorithm to analyze its foreshock and aftershock sequences. Detailed links and family structures of the sequence are obtained. The results show that a *M*_W_5.0 event at 03:16 (UTC) on 6 July is a direct foreshock of the *M*_W_7.1 mainshock. It is likely related to barriers on the northwest-striking fault. The *M*_W_6.4 event on 4 July is characterized as a complex conjugate rupture. Notably, a magnitude 4.0 event occurred on the northwest-striking fault before the *M*_W_6.4 event, establishing it as a direct foreshock. The Ridgecrest sequence is predominantly influenced by northwest fault activity. It first caused small fractures on the northwest-striking fault. Then, it triggered conjugate slips on the southwest-striking fault. Lastly, it led to larger ruptures on the northwest-striking fault.

## 1. Introduction

The pattern and frequency of earthquake sequences can vary based on the tectonic settings, the size of the mainshock, and the characteristics of the fault system involved. Understanding earthquake sequences is important for assessing seismic hazards and preparing for potential future events. The July 2019 Ridgecrest *M*_W_7.1 earthquake in Southern California initiated a unique sequence, resulting in numerous aftershocks as well as notable foreshocks, which highlights the intricate rupture process along conjugate structures [1,2,3].

Similarly to other large earthquakes (e.g., 2010 Yushu *M*_W_7.1 [4], 2011 Tohoku *M*_W_9.0 [5], and 2014 Chile *M*8.1 [6]), 2019 Ridgecrest *M*_W_7.1 had a foreshock with *M*_W_6.4 [7]. Foreshocks have always been the focus of seismologists, but they are still an ambiguous concept without an accurate definition [8,9]. Research into foreshocks and their role in rupture history can help us to make progress in earthquake prediction [10,11,12], to analyze the triggering process [13,14,15], and to understand the implications for earthquake nucleation [16,17,18,19,20].

There are many works that have studied the relationship between the mainshock and the foreshock. Researchers hope to approach earthquake forecasting through the statistical characterization of foreshocks [21]; Helmstetter et al. [22] suggest that mainshocks are aftershocks of conditional foreshocks; and Lippiello et al. [23] discuss the relevance of foreshocks in the triggering of earthquakes from a statistical viewpoint. Petrillo et al. [24] investigate the influence of the brittle–ductile transition zone on aftershock and foreshock occurrence.

Research on foreshocks is topical, especially with new technologies. For example, using the same high-resolution seismic catalog enhanced by the template matching technique, Trugman and Ross [25] suggest that foreshocks existed in 72% of the mainshocks in Southern California, whereas van den Ende and Ampuero [26] argue that this value should be less than 18%. Moutote et al. [27] use the ETAS model and find that 10/53 mainshocks were preceded by significant foreshocks in the same area. Wetzler et al. [28] systematically examine foreshocks via different approaches.

The 2019 Ridgecrest *M*_W_7.1 earthquake sequence provides great opportunities to study foreshocks. In this paper, we focus on the relationship between the foreshock sequence and the 2019 Ridgecrest *M*_W_7.1 mainshock. Based on the relocated catalogs, we apply the nearest neighbor distance algorithm to analyze the intrinsic spatiotemporal structure of this significant foreshock sequence. This algorithm is widely used and has proven effective in clustering analysis for earthquakes. We also examine its statistical features. This work might help us to understand the complex dynamic relationships in earthquake sequences and provide some clues and insights into the related focus mechanisms.

## 2. Theories and Methods

There are many cluster analysis methods in seismology [29,30,31,32,33,34]. To study the Ridgecrest sequence, we adopted the so-called “nearest neighbor distance” (NN) method [35], which was developed in recent years [36,37,38].

A more complex approach to distinguishing clustering events from background events involves assigning a generalized distance between the shocks. The subjective aspect in this process involves choosing a formula for distance and a threshold to define independent events. Usually, the distances in the time, space, and energy domains are considered separately. The challenge is how to put them all together with more physical meanings. The NN algorithm proposed by Zaliapin et al. [36] comprehensively considers the distribution of earthquakes in the space, time, and energy domains. It also provides a normalized parameter that makes the complex N-dimensional problem easier to handle. Based on this defined distance, we can examine the relationship between any two earthquakes and distinguish clustering events from background earthquakes.

The application of this method in various regions worldwide has demonstrated its effectiveness, e.g., Moradpour et al. [39] in Southern California, Peresan and Gentili [40] in Italy, Bayliss et al. [41] in Japan and New Zealand, Martínez-Garzón et al. [42] in Turkey, and Zaliapin and Ben-Zion [43] for the whole world.

A brief description of the NN algorithm is given below. Each event in the catalog is characterized by 5 numbers: {t,λ,ϕ,h,m} (time, latitude, longitude, depth, magnitude). For a given earthquake catalogue $\{t_{i},\lambda_{i},\phi_{i},h_{i},m_{i}\},i=1,\cdots ,N$, the asymmetric distance $n_{ij}$ from the event $\left\{t_{j},\lambda_{j},\phi_{j},h_{j},m_{j}\right\}$ to another event $\left\{t_{i},\lambda_{i},\phi_{i},h_{i},m_{i}\right\}$ is defined by the following formula [36]:

(1) $$n_{ij}=\left\{\begin{array}{c} c\tau_{ij}r_{ij}^{d}10^{-b(m_{i}-m_{0})}\\ \infty \end{array}\right.\begin{array}{c} \tau_{ij}\geq 0\\ \tau_{ij}<0 \end{array},$$

where $\tau_{ij}=t_{j}-t_{i}$, $r_{ij}$ is the geographical distance between two events, $m_{0}$ is the magnitude of the lower threshold of the catalog, c is the constant coefficient, d is the fractal dimension of earthquake epicenters, and b is the exponent in the Gutenberg–Richter (GR) law.

Each event $\left\{t_{i},\lambda_{i},\phi_{i},h_{i},m_{i}\right\}$ (except for the first event in the catalog) has one closest “ancestor”—that is, among the preceding events, the one that is the shortest distance away from it. This is called its nearest neighbor. The minimum distance to the nearest neighbor is

(2) $$\eta_{j}^{*}=\min_{i}n_{ij},$$

We performed some numerical tests for synthetized and real catalogs, and the results proved that the NN algorithm can distinguish clustering events from background activities more physically [44]. Based on the nearest neighbor distances of events, we can classify the type of events in the whole sequence—statistically, foreshocks, mainshocks, and aftershocks.

**Figure 1:**
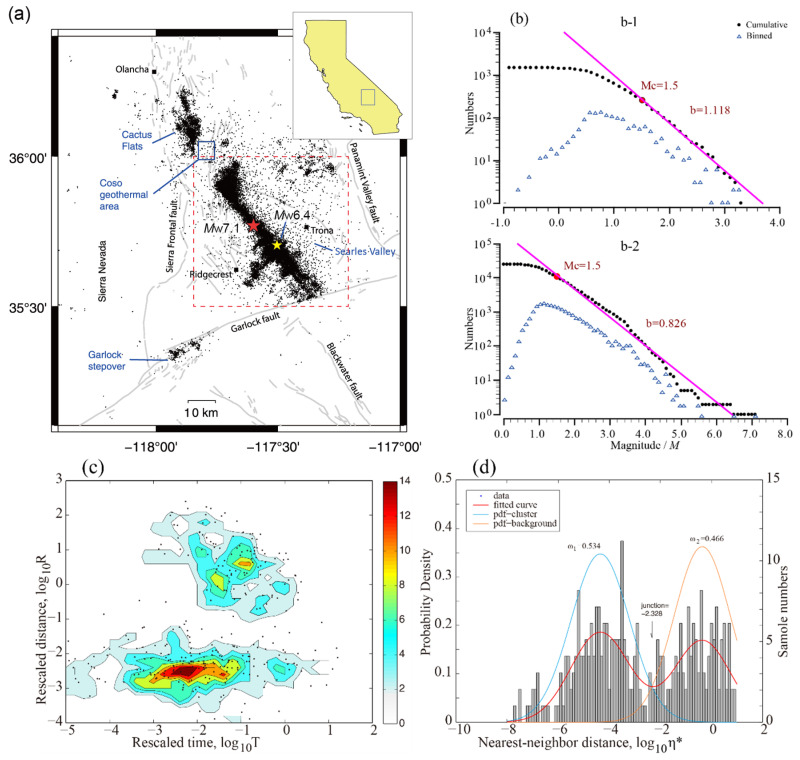
(**a**) Distribution map of the 2019 Ridgecrest sequence and regional seismic activities. The dashed red rectangle denotes the select range of the Ridgecrest sequence, which is studied next in this paper. Events that occurred in Cactus Flats and the Coso geothermal area are not included in the sequence, mainly because the activities in these areas have been proven to be non-tectonic earthquakes. (**b**) The Gutenberg–Richter (GR) relationship for (**b-1**) regional activities in the first half of 2019 and (**b-2**) the 2019 Ridgecrest sequence. Solid purple lines show the fitting results. (**c**,**d**) Results of the NN algorithm for the catalog of (**b-1**): (**c**) 2D distribution and density contours of re-scaled time (tj) and re-scaled distance (rj) components; (**d**) histogram of re-scaled nearest neighbor distances η* for the ECSZ *M*1.5+ catalogue in the first half of 2019, with the red solid curve denoting the fitted probability density function (PDF). The azure bell-shaped curve represents the clustering part, while the orange bell-curve represents background events.

**Figure 2:**
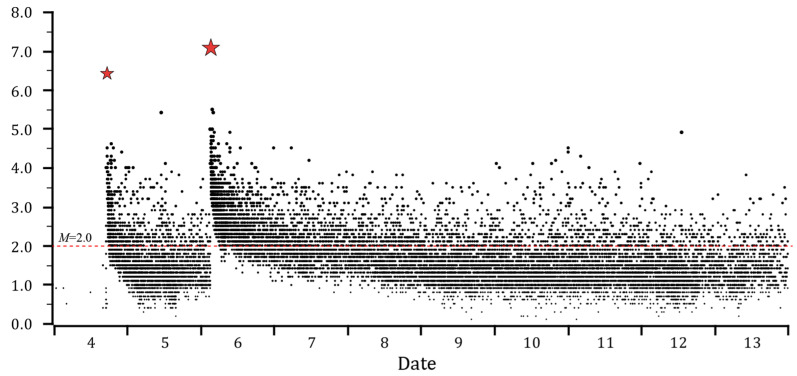
Magnitude–time plot for the first week of the Ridgecrest sequence. The two red stars denote the *M*_W_7.1 mainshock and the *M*_W_6.4 foreshock, respectively. Note the catalog’s incompleteness during the short time after the large events and the activities before the *M*_W_6.4 foreshock. The red dashed line denotes *M* = 2.0, and the catalog above this limitation seems more complete.

It is possible to further define the temporal and spatial components of the magnitude-normalized NN distance [36]:

(3) $$T_{ij}=\tau_{ij}10^{-bm_{i}/2},\;R_{ij}=r_{ij}^{d}10^{-bm_{i}/2},$$

and n=TR (without loss of generality, it is assumed here that $c=1,m_{0}=0$). We therefore can analyze spatiotemporal features via distribution plots of *T* and *R*. Two-dimensional images always provide more details than 1D images, and therefore we can obtain some additional statistical information, as discussed by Li et al. [45]. This can be helpful for further analysis (see Figure 1c in this paper or, for example, Figure 4 in [37,43] and Figures 2 and 4 in [44]).

Previous works usually used images for simple statistical or qualitative evaluation, and lacked further quantitative analysis. Since the bimodal distribution of nearest neighbor distances has been confirmed by many researchers, Peresan and Gentili [40] estimated Gaussian densities for clustered and background components, and Bayliss et al. [41] applied the Markov chain Monte Carlo approach to build normal mixture models. In addition, we also used a Gaussian mixture model to fit 2D and 1D distributions [44]. Assuming that clustering and background earthquakes both satisfy the Poisson process with different parameters, we can transform the 2D superimposed Poisson process into a 1D mixture density function referring to the concept of the N-th order distance. We define the mixture density function for the nearest neighbor distance η* as

(4) $$PDF=\omega_{1}\cdot pdf_{1}+\omega_{2}\cdot pdf_{2}$$

where *pdf*_1_ and *pdf*_2_ are probability density functions of clustering and background events, respectively, both for a normal distribution, while *ω*_1_ and *ω*_2_ are proportionality coefficients, which satisfy *ω*_1_ + *ω*_2_ =1. When we calculate the nearest neighbor distances η* for a catalog, we can fit its probability distribution according to Formula (4). Then, we can distinguish clustering events from background activities.

## 3. Data and Study Area

At 3:19 (UTC, the same below) on 6 July 2019, an *M*_W_7.1 earthquake occurred in the desert about 10 km northeast of Ridgecrest, Southern California, while a *M*_W_6.4 foreshock occurred near the epicenter about 34 h before this earthquake, that is, at 17:33 on 4 July 2019. The *M*_W_7.1 mainshock occurred in the NW–SE-trending fault zone with a dextral strike-slip trend, while the *M*_W_6.4 foreshock occurred in the NE–SW-trending fault. The aftershocks of the *M*_W_7.1 mainshock spread in the NW–SE direction, extending northward to China Lake and southeastward to the Garlock fault, but did not cross this fault. The mainshock was followed by more than 100,000 aftershocks. This Ridgecrest earthquake sequence was systematically observed and well documented due to the high density of instruments in California [1].

The epicenter of this earthquake was located in the Eastern California Shear Zone (ECSZ), about 150 km northeast of the San Andres Fault and about 12 km southwest of the Searles Valley in California (Figure 1a). The Eastern California Shear Zone is a seismically active region characterized by strike-slip faults, with a historical record of numerous earthquakes exceeding magnitude 7, including the Landers *M*_W_7.1 earthquake in 1992 and the Hector Mine *M*_W_7.1 earthquake in 1999. However, since 2000, the seismic activities in this area have been mainly composed of small and medium-scale events (only 3 events with *M* ≥ 5.0).

The catalog before July 2019 is from the Southern California Seismic Network (SCSN), which is available online (https://scedc.caltech.edu/eq-catalogs/index.html, accessed on 30 October 2024). Figure 1b gives the frequency–magnitude distributions and their fitted lines, (b-1) for this catalog and (b-2) for the Ridgecrest sequence. In the first half of 2019, there were about 690 events with *M* ≥ 1.0 recorded by SCSN, and the average *b* = 1.118. The higher *b*-value we obtained is close to the previous estimate for seismicity in the Coso Range (0.99 ± 0.08, from [46]). The lowest magnitude of completeness in this area then can be derived from the fitted line, *M*c ≈ 1.5. We then selected *M* ≥ 1.5 events from the catalog and applied the NN algorithm to study its clustering features. The bimodal distribution of nearest neighbor distances is clearly shown in Figure 1c. The horizontal spreading part is for clustering events, and the skewed part on the upper right panel is for background events. The histogram and its fitted probability density function (PDF) in Figure 1d confirm the bimodal distribution. We can derive from the fitted result that *ω*_1_ = 0.534 and *ω*_2_ = 0.466, implying a higher clustering events ratio in regional seismic activities. As mentioned previously, the local seismicities in the Coso area are mainly characterized by sequences and small swarms [46]. The clustering events are independent of background events, this is in agreement with previous studies [47,48]. Here, we give the intersection point of the two PDFs, *J*x = −2.328—that is to say, the probability for an event to be background or belong to clusters at this point is equivalent. If the nearest neighbour distance η* of any event is smaller than *J*x, the event is more likely to be clustered.

The accurate location of earthquakes is helpful for depicting the spatial and temporal distribution images in more detail for earthquakes in the source area and determining the spatial distribution of seismic faults [49]. By using data from densely deployed arrays and the Southern California Seismic Network (SCSN), White et al. [49] derived a more precise earthquake catalogue for the 2019 *M*_w_ 6.4 and *M*_w_7.1 Ridgecrest earthquake sequence. We use this catalogue in our study for further nearest neighbor analysis. We analyze the temporal and spatial distribution characteristics of the hypocenters and discuss the intrinsic relationship among events in this sequence.

Based on White et al.’s catalog [49], we selected the events within the red rectangle in Figure 1a for our next analysis. The clusters that occurred in Garlock stepover, as well as in Coso geothermal field and Cactus Flats (see in Figure 1a), which were clearly identified as induced activities (as studied in many papers, e.g., [2,50]), were not included in the sequence. We first determined the lowest magnitude of completeness *M*c of the sequence based on the GR relationship. Figure 1b shows the results of the least square method: the *b*-value of the Ridgecrest sequence is about 0.826, and the approximate magnitude of completeness *M*c ≈ 1.5. However, it is obvious from the fitting result in Figure 1(b-2) that the linear relationship does not match the data well. The reason for this situation, as shown in Figure 2 (and also analyzed in [1]), would be that small-magnitude events following a large earthquake are missing at the very beginning point, even when the template matching technique is performed. We thus selected events with M ≥ 2.0 for subsequent calculations, just like the criteria in Hardebeck’s study [2]. In order to reduce the amount of computation, we chose 7 days of data after the mainshock for nearest neighbor calculation, referring to Chen’s study [51].

## 4. Results

### 4.1. Family Relations in the Whole Sequence

**Figure 3:**
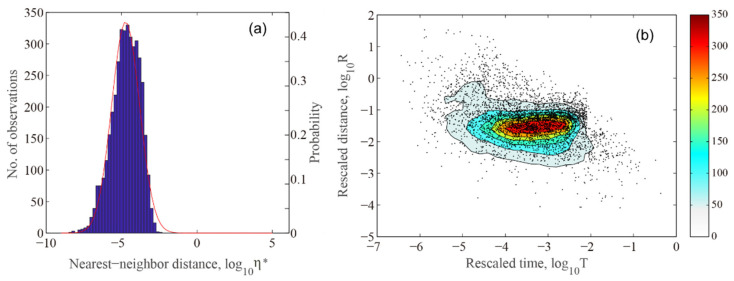
Statistical results of the nearest neighbor distance η* for the Ridgecrest *M*_W_7.1 earthquake sequence. (**a**) Nearest neighbor distance η* statistical histogram with normal distribution fit; (**b**) contour plot of the two-dimensional spatiotemporal distribution density.

We applied the NN algorithm to analyze family relations in the 2019 Ridgecrest earthquake sequence. From the histogram of its nearest neighbor distance η* (Figure 3a), it can be fitted by a single normal probability density function, which implies that most events in the Ridgecrest earthquake sequence belong to clustering earthquakes. This opinion also can be confirmed by the spatiotemporal distribution of η* (Figure 3b). The data encircle around a horizontal line, as discussed by other researchers [38,45], which indicates that aftershocks are restricted in a limited spatial area and extend only to the time domain.

**Figure 4:**
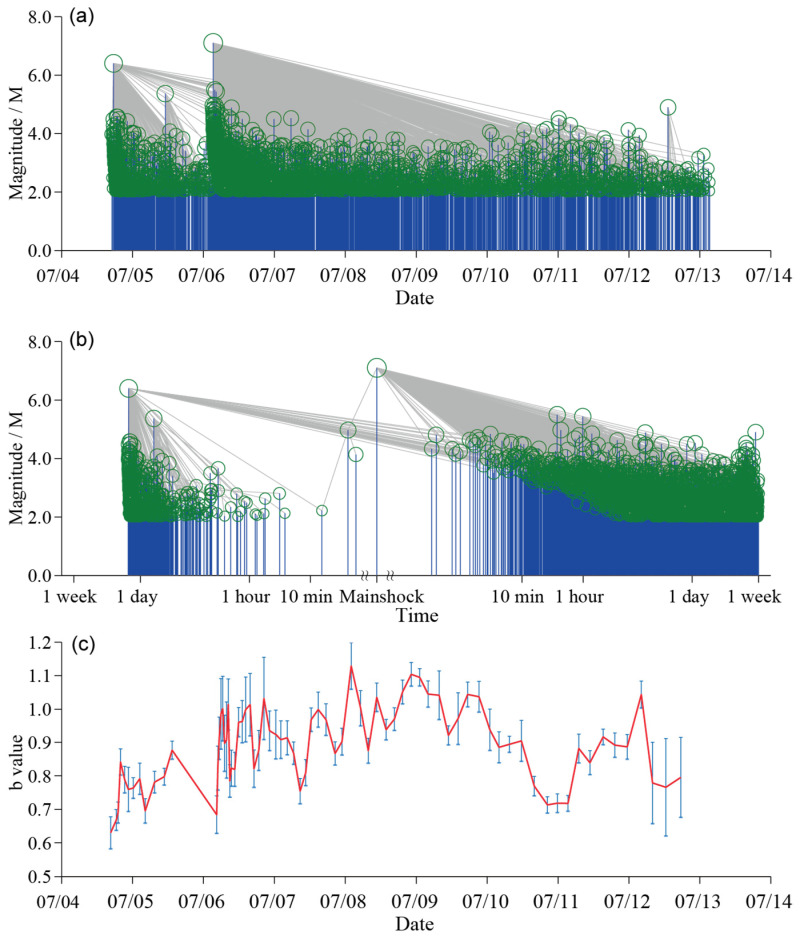
Magnitude–time plot of *M* ≥ 2.0 events in the Ridgecrest sequence (**a**). Panel (**b**) is the same as (**a**), but the logarithmic time coordinate is applied. Green circles and blue lines in (**a**,**b**) denote earthquakes, while gray lines are links. (**c**) shows the time variation of the *b*-value.

We connect each *M* ≥ 2.0 event in the Ridgecrest sequence to its nearest neighbor (as in Figure 2 in [38]), which can be seen as its parent event, according to the distance η* (see Figure 4). The logarithmic coordinate is applied on the horizontal time axis in Figure 4b to demonstrate the structures of the foreshock sequence more clearly.

It is obvious that the *M*_W_7.1 mainshock plays a dominant role in the aftershock activities of the sequence. Most aftershocks are physically intimately related to it and are its offspring events. However, its influence gradually weakens with passage of time. After a few days, some larger aftershocks also have their own aftershocks (Figure 4a). It is also noted in Figure 4 that the impact of the *M*_W_6.4 foreshock does not vanish with the occurrence of the *M*_W_7.1 mainshock. There are still many aftershocks that can be seen as offspring of the *M*_W_6.4 event in the sequence after the *M*_W_ 7.1 mainshock. This is also consistent with other studies [3].

The changes in *b*-value in the G-R relationship demonstrate variation in tectonic stress. Figure 4c shows the *b*-value time series along with the Ridgecrest sequence. Here, the *b*-values were calculated with equal event numbers; specifically, the window size is 240, and the step size is 30. The *b*-value increases within the first few days. At the start of the sequence, the *b*-value is significantly lower (<0.7) and then rises to nearly 0.8. Throughout the entire foreshock sequence, the *b*-values remain below 0.8. Aftershocks following the *M*_W_7.1 mainshock showed elevated *b*-values compared to foreshocks, yet remained below background levels (1.118, as mentioned above). This is consistent with previous results from other studies [52]. The foreshock sequences always have lower *b*-values [53], as observed before many large earthquakes (e.g., [54,55,56]), as well as in laboratory observations [57]. This seismic pattern reveals shifting stress dynamics throughout the earthquake sequence, hinting at complex tectonic interactions beneath the surface.

### 4.2. M_W_6.4 Foreshock Sequence

The *M*_W_6.4 foreshock occurred in ECSZ on the side of the California plate boundary. The rupture began on a northwest-striking right-lateral fault and then continued on a southwest-striking fault with mainly left-lateral slip [3,58]. The *M*_W_6.4 foreshock sequence might have triggered the *M*_W_7.1 Ridgecrest mainshock. However, if we refer to the nearest neighbor link in Figure 4, the *M*_W_6.4 event is probably not the immediate cause of the rupture of the *M*_W_7.1 mainshock. From Figure 4a,b, the parent event of the Ridgecrest *M*_W_7.1 mainshock, or, in other words, the direct foreshock, is an *M*_W_5.0 event that occurred at 03:16 a.m., which is about 3 min and 20 s before the mainshock. This is the ‘ancestor’ event to the mainshock by means of statistical significance. We then examine the activities of the *M*_W_6.4 foreshock sequence.

**Figure 5:**
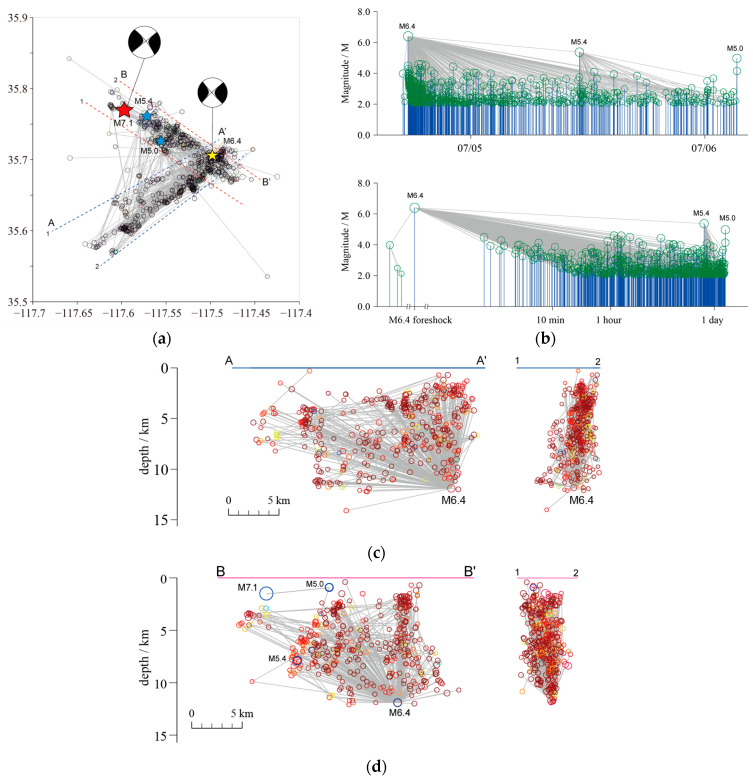
Distribution of the *M*_W_6.4 foreshock sequence. (**a**) shows the epicenters of the sequence, where the red star denotes the *M*_W_7.1 mainshock, while the yellow star is the *M*_W_6.4 foreshock, and the blue stars indicate two *M* ≥ 5 events; (**b**) shows the NN results for *M* ≥ 2.0 events in the *M*_W_6.4 foreshock sequence, here the *M*_W_6.4 event is seen as a mainshock. Green circles and blue lines denote earthquakes, while gray lines are links. Events with epicenters located between two NE–SW-trending solid blue lines are projected on an AA’ profile (**c**), and (**d**) is the profile of events between two dashed red lines. Different colors of circles in panel (**c**,**d**) denote the time order of these events.

The relocated catalogue of the *M*_W_6.4 foreshock sequence shows that the rupturing process can be divided into two parts: a northwest-striking fault and a southwest-striking fault (see Figure 5a). Notably, all the *M* ≥ 5.0 events occurred on the northwest-striking fault, suggesting that this fault is the main rupturing fault.

It can be derived from Figure 5c,d that the *M*_W_6.4 event invoked aftershocks in both faults. But, compared to the northwest-striking fault (BB’), the cracks were more evenly distributed on the southwest-striking fault’s plane (AA’). The biggest aftershock of the *M*_W_6.4 foreshock was a *M*_W_5.4 event. This event and its own aftershocks were clustered on the northwest-striking fault (BB’). In the left section of Figure 5d (i.e., in the northwest direction of the *M*_W_6.4 event), all aftershocks, except a small group, appear to be halted by a line, particularly in the shallower areas. These phenomena of earthquake migration and seismicity quiescence have already been discussed in [59]. This indicates that there might be a barrier on the fault. The *M*_W_5.4 event and its own aftershocks took place at the edge of the barrier, but did not break it. Finally, the *M*_W_5.0 event took place at the end part of the quiescence area, and then the *M*_W_7.1 mainshock.

In other words, the *M*_W_5.4 event is classical aftershock of the *M*_W_6.4 event, while the *M*_W_5.0 event, which occurred after the *M*_W_6.4 and *M*_W_5.4 events, is not aftershock of either of them. It has a much closer relationship with the *M*_W_7.1 mainshock (as can also be seen in Figure 4b). We finally give the nearest neighbor for *M* ≥ 5.0 events in the 2019 Ridgecrest *M*_W_7.1 sequence (Table 1).

### 4.3. Foreshocks of the M_W_6.4 Foreshock

**Figure 6:**
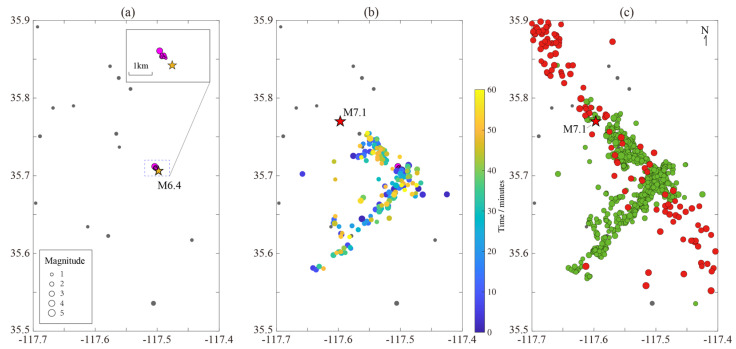
The distribution maps of earthquakes in different periods in the Ridgecrest area. (**a**) Gray dots are earthquakes that occurred in June 2019, and filled purple circles are earthquakes that occurred from 1st July to 17:33 on 4 July, before the *M*_W_6.4 event in the Ridgecrest sequence. The small figure in the upper right corner is an enlargement of the dashed box in this panel; (**b**) circles filled with a gradually changing color from blue to yellow are aftershocks which occurred in the 1 h after the *M*_W_6.4 event; (**c**) green circles represent the *M*_W_6.4 foreshock sequence, while red circles represent aftershocks in the 1 h after the *M*_W_7.1 mainshock. Note that the whole *M*_W_6.4 foreshock sequence did not expand any more than its beginning time, as shown in panel (**b**).

We examined the catalogues from White et al. [49] and SCSN. No earthquakes took place in the epicenter area of the oncoming *M*_W_6.4 event, at least in June (Figure 6a). Moving into July, two small events with *M* = 1.5 occurred on 1st July in the rupture area of the coming *M*_W_7.1 earthquake. So, what is the relationship between these two microshocks and the *M*_W_6.4 or *M*_W_7.1 event? Are they the pre-ruptures of the fault? From our calculation, the distance between the latter one and its nearest events in the Ridgecrest sequence η*≈3.343, which is much larger than the *J*_X_ = −2.328 in Figure 1. This indicates that these two weak events do not belong to clustering events, and therefore should be seen as background activities. Another piece of evidence is that there were also two *M* > 1.0 events almost at the same location in January 2019. We therefore did not include them in the Ridgecrest sequence.

**Figure 7:**
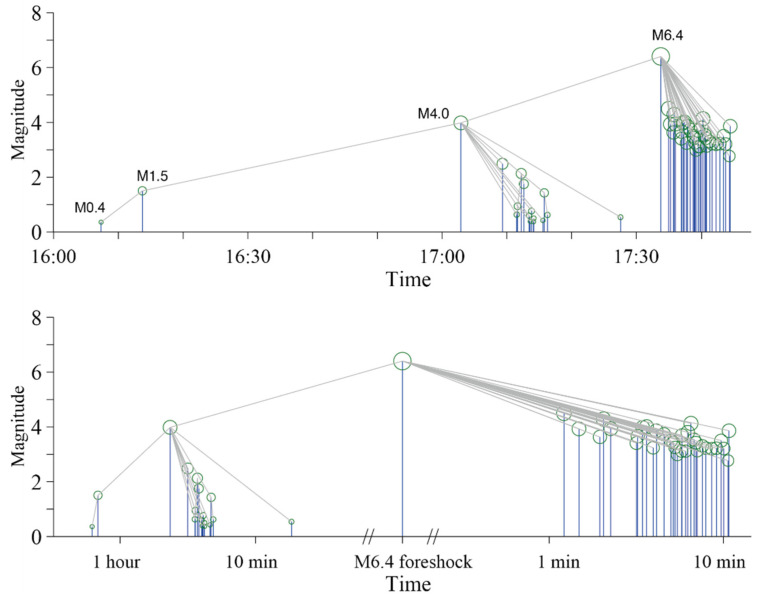
Family links of the events before and after the *M*_W_6.4 foreshock. Green circles and blue lines indicate earthquakes and gray lines correspond to parent links. The **upper** panel shows the family links of magnitude over time. The **lower** panel is almost the same, but the horizontal coordinate is in logarithmic scale.

Following this, 3.5 days later, the Ridgecrest sequence begins. A small set of weak earthquakes arrived ahead of the *M*_W_6.4 event, with the biggest *M* = 4.0 event being at 17:02 on 4 July (Figure 6a). From the NN algorithm (Figure 7), the *M*4.0 earthquake is the parent event of the *M*_W_6.4 earthquake. Statistically, the *M*4.0 event at 17:02 is the foreshock of the *M*_W_6.4 event, while the *M*1.5 event at 16:13 is the foreshock of the *M*4.0 event, and the *M*0.4 event at 16:07 is the foreshock of *M*1.5 event.

The *M*_W_6.4 earthquake is seen as conjugate faulting [1,50,60,61]. In the foreshock sequence, the *M*_W_6.4 event triggers simultaneous activities in both the northeast and northwest faults. As shown in Figure 6b, aftershocks form a ‘T’ shape just minutes after the *M*_W_6.4 event. Subsequent activities remained within this area until the *M*_W_7.1 mainshock outbreak (Figure 6c). The *M*4.0 foreshock sequence extended in the northwest direction (Figure 6a). Our analysis suggests that the initial rupture of the *M*_W_6.4 event likely originated from the northwest faults. This is also in agreement with other studies [51,60,61].

## 5. Conclusions and Discussion

Large earthquakes can occur in a more complex fault system than commonly assumed. A series of ruptures occur in a web of interconnected faults, with rupturing faults triggering other faults. The stress changes and interactions during the earthquake sequence are intricate and complicated. The 2019 Ridgecrest *M*_W_7.1 earthquake sequence occurred in these exact tectonic conditions. In addition to numerous studies on this sequence based on many aspects, e.g., focus kinematics and dynamics, this paper employs statistical techniques to elucidate the relationships between each event within it.

The Ridgecrest *M*_W_7.1 earthquake has a foreshock sequence characterized by a significant *M*_W_6.4 event. However, the results presented in this paper indicate that the *M*_W_6.4 event is not the foreshock of the *M*_W_7.1 mainshock, as is generally supposed. Instead, a *M*_W_5.0 event in the foreshock sequence, which occurred about 3 min and 20 s before the *M*_W_7.1, is the direct foreshock of the mainshock. This event occurred in a quiescence area along the northwest-striking fault in the foreshock sequence, and also with a shallower focus, just like the mainshock. It was located about 6km southeast of the initial rupture point of *M*_W_7.1 mainshock. We suppose that this mid–strong event may be linked to barriers that generated the *M*_W_7.1 mainshock.

The *M*_W_6.4 event triggered aftershocks in both the NW- and SW-direction faults, even several minutes after it. The SW-striking fault ruptured fairly significantly, while subsequent aftershocks on it did not exceed the initial range formed at the beginning. In contrast, the ruptures on the NW-striking fault seem to be restricted or hindered. With the development of the *M*_W_6.4 sequence, aftershocks gradually extended beyond its initial limitations in the NW direction, ultimately leading to the occurrence of the *M*_W_7.1 mainshock.

Before the *M*_W_6.4 earthquake, there was still a small group of events in the sequence. The biggest event in the sequence is a *M*4.0 medium-strength earthquake, which occurred about 30 min before the *M*_W_6.4 event. This small cluster occurred at a place between the *M*_W_7.1 and *M*_W_6.4 events, northwest of *M*_W_6.4 and also on the NW-striking fault. From this paper, the *M*4.0 event is the direct foreshock of *M*_W_6.4. This implies that NW fault activity dominates the whole sequence. Small fractures emerge on the NW faults and then trigger conjugated fault slips. Barriers on the NW faults retard the development of ruptures, and a much larger rupture takes place at last.

Based on the nearest neighbor distance algorithm, we analyze the rupture process of the 2019 Ridgecrest sequence. Our findings lead to similar conclusions to those in works focused on other aspects. These findings indicate that statistical approaches with physical meanings can also receive the same understanding in seismology. The results shown in this paper are only from statistical analysis. As demonstrated by other works, foreshocks might have lower stress drops [14,62], higher waveform similarity [57], and other source features [63,64,65]. We hope that the direct ‘foreshocks’ recognized in this paper may have some different features which can be derived from their waveforms or other observations. Multidisciplinary studies and more elaborate works on this topic are thus required in the future.

## Figures and Tables

**Table 1:** Nearest neighbors of *M* ≥ 5.0 events in the 2019 Ridgecrest sequence.

Event No.	*M* ≥ 5.0 Earthquakes (yyyy-mm-dd HH:MM:SS.s)	Nearest Neighbours
1	2019-07-04 17:33:48.6 *M*_W_6.4	2019-07-04 17:02:55.4 *M*4.0
2	2019-07-05 11:07:53.2 *M*_W_5.4	2019-07-04 17:33:48.6 *M*_W_6.4
3	2019-07-06 03:16:32.1 *M*_W_5.0	2019-07-06 03:12:41.0 *M*2.2
4	2019-07-06 03:19:52.3 *M*_W_7.1 ^1^	2019-07-06 03:16:32.1 *M*_W_5.0
5	2019-07-06 03:47:53.0 *M*_W_5.5	2019-07-06 03:19:52.3 *M*_W_7.1 ^1^
6	2019-07-06 04:18:55.4 *M*_W_5.4	2019-07-06 03:19:52.3 *M*_W_7.1 ^1^

^1^ Mainshock.

## Data Availability

Third-part data.
